# Identification of potential hub genes and drugs in septic liver injury: A bioinformatic analysis

**DOI:** 10.1097/MD.0000000000049060

**Published:** 2026-06-19

**Authors:** Yan Yang, Jing Lv, Jianfeng Chu, Guobin Song, Shujun Sun, Rui Chen

**Affiliations:** a Department of Anesthesiology, The First People’s Hospital of Jiangxia District, Wuhan, China; b Department of Anesthesiology, Union Hospital, Tongji Medical College, Huazhong University of Science and Technology, Wuhan, China; c Department of Anesthesiology, Shannan Maternal and Child Health Hospital, Shannan, Xizang, China; d Department of Anesthesiology, Zhejiang Hospital, Hangzhou, China.

**Keywords:** bioinformatics analysis, drug discovery, legionellosis and cellular response to lipopolysaccharide, malaria, septic liver injury, text mining

## Abstract

**Background:**

The liver is pivotal in the metabolic and innate immune responses of sepsis, managing bacteremia, cytokine regulation, and acute-phase protein synthesis. However, the liver’s susceptibility to damage during sepsis underscores the need to understand the mechanisms behind septic liver injury. Our objective was to apply bioinformatics to identify key genes and pathways involved in septic liver injury and to reveal potential therapeutic targets.

**Methods:**

We utilized pubmed2ensembl to identify genes associated with septic liver injury and performed functional annotation and pathway analysis using Xiantao. Protein-protein interactions were analyzed via the STRING database, and hub genes were identified with Cytoscape software. Candidate genes were validated with Metascape, and drug-gene interactions were explored using DGIDB.

**Results:**

Our analysis identified 63 genes implicated in sepsis-associated liver injury, refining our understanding of its molecular landscape. Gene ontology and Kyoto encyclopedia of genes and genomes analyses shortlisted 42 candidate genes, highlighting their roles in septic liver injury pathogenesis. An PPI network analysis extracted 18 genes, with MCODE-assisted analysis revealing a module of 8 key genes central to septic liver injury pathophysiology. These genes – TLR4, CXCL8, IL-18, IL-6, IL1B, tumor necrosis factor, NFKB1, and colony-stimulating factor 3 – are linked to 3 major signaling pathways: malaria, legionellosis, and the cellular response to lipopolysaccharide. Furthermore, 23 drugs targeting these genes were identified, suggesting their potential as therapeutic agents for septic liver injury.

**Conclusion:**

The genes TLR4, CXCL8, IL-18, IL-6, IL1B, tumor necrosis factor, NFKB1, and colony-stimulating factor 3 are central to the pathology of septic liver injury, acting as critical mediators of associated inflammatory processes. The correspondence of these genes with 23 drugs demonstrates their therapeutic potential, elucidating molecular targets for future interventions and paving the way for novel treatment strategies. This study provides a robust framework for subsequent research endeavors and the development of targeted therapies, enhancing our capacity to address septic liver injury effectively.

## 1. Introduction

Sepsis, a systemic inflammatory response syndrome triggered by infection, can lead to organ dysfunction and pose a life-threatening risk. The liver, integral to the immune system and metabolic processes, is an early indicator of adverse outcomes in sepsis.^[1]^ Approximately 30% of sepsis cases exhibit liver injury. During acute liver failure, extensive hepatocyte necrosis occurs, accompanied by the activation of immune complexes that trigger the complement system and the release of pro-inflammatory cytokines like tumor necrosis factor-alpha (TNF-α) and interleukin-6 (IL-6), leading to a cascade of inflammatory responses.^[2]^ Mitigating liver injury may decrease the incidence and mortality rates among sepsis patients. The pathogenesis of septic liver injury is multifaceted, involving complex interactions among various factors, and currently lacks a definitive treatment. Identifying target genes and therapeutic agents is crucial for predicting and treating septic liver injury.

The proliferation of scientific literature presents a formidable challenge in tracking the latest research findings manually, and necessitates the use of text-mining methods to identify these interactions. Text mining methods offer a solution by extracting pertinent knowledge from scientific literature.^[3]^ The effective clustering of functional genes, mainly based on gene ontology (GO) and kyoto encyclopedia of genes and genomes (KEGG) pathway, can also help solve these problems. These pathways cluster functional genes into different biological processes.^[4]^ The Drug-Gene Interaction Database aggregates data from publications, elucidating the connections between drug and medicinal genes and their potential applications in therapy.^[5]^

In this study, we harnessed text mining, functional and pathway analysis, and database interrogation techniques to identify a plethora of potential genes and extant drugs for the amelioration of infectious liver injury. We first obtained a preliminary list of relevant genes when exploring potential drugs for treating infectious liver injury. Subsequently, we integrated the gene data through functional and pathway enrichment analyses, culminating in a roster of high-priority target genes. Ultimately, we derived candidate drugs from the analysis of drug-gene interaction datasets, providing a foundation for targeted therapeutic development.

## 2. Methods and materials

### 2.1. Data sources

A gene expression profile (GSE226278) of septic liver injury disease was downloaded from the GEO database (https://www.ncbi.nlm.nih.gov/gds/?term=GSE226278). All data are publicly available for online download.

### 2.2. Identification of differentially expressed genes

GSE226278 dataset was downloaded from GEO and analyzed using R, dividing the data into SLI and control groups, with “*P* < .05 and log (fold change) >1 or log (fold change) <−1” defined as screening for differential genes (DEGs) thresholds.

### 2.3. Text mining

For text mining endeavors, the pubmed2ensembl tool was engaged (http://pubmed2ensembl.ls.manchester.ac.uk/), with “Homo sapiens” specified as the focal species. The term “septic liver injury” was applied to filter pertinent literature, yielding a curated gene list.

### 2.4. GO biological process and KEGG pathway analyses

The genes culled from text mining were subjected to GO and KEGG pathway analyses to delineate their biological roles and pathways. Xiantao (https://www.xiantao.love/) was harnessed for visual representation of the data.

### 2.5. Protein–protein interactions

STRING database (http://stringdb.org), a curated repository of known and predicted protein interactions, was utilized to delineate the network of interactions among the 42 candidate genes identified from the functional enrichment analysis. The gene list was input into STRING, which automatically maps gene symbols to their corresponding protein entities based on its integrated database. To ensure the reliability of the interactions, a confidence score threshold of medium confidence (0.400) was applied to filter out low-probability interactions. Furthermore, to focus the analysis on biologically meaningful connections, the option to hide disconnected nodes (proteins that lack any interaction partners meeting the confidence threshold) was enabled during network construction. This approach prioritizes the analysis of interconnected protein modules with higher biological relevance.

### 2.6. Network analysis with cytoscape

Cytoscape, a versatile platform for network analysis, was employed to manage and visualize the protein-protein interaction data.^[6]^ The MCODE plugin facilitated the identification of highly interconnected regions and potential hub genes.^[7]^

### 2.7. Functional enrichment of hub genes

Metascape (https://www.metascape.org), an online analytical suite, was utilized for the functional annotation and enrichment analysis of the hub genes, with a significance threshold of *P* <.05.^[8]^

### 2.8. Drug-gene interactions

The Drug-Gene Interaction Database (DGIdb, www.DGIdb.org) provided insights into the interactions between drugs and genes.^[5]^ A stringent filter was applied, requiring a query score >4 and an interaction score >2, to identify candidate drugs with potential therapeutic relevance to septic liver injury.

**Figure 1.**
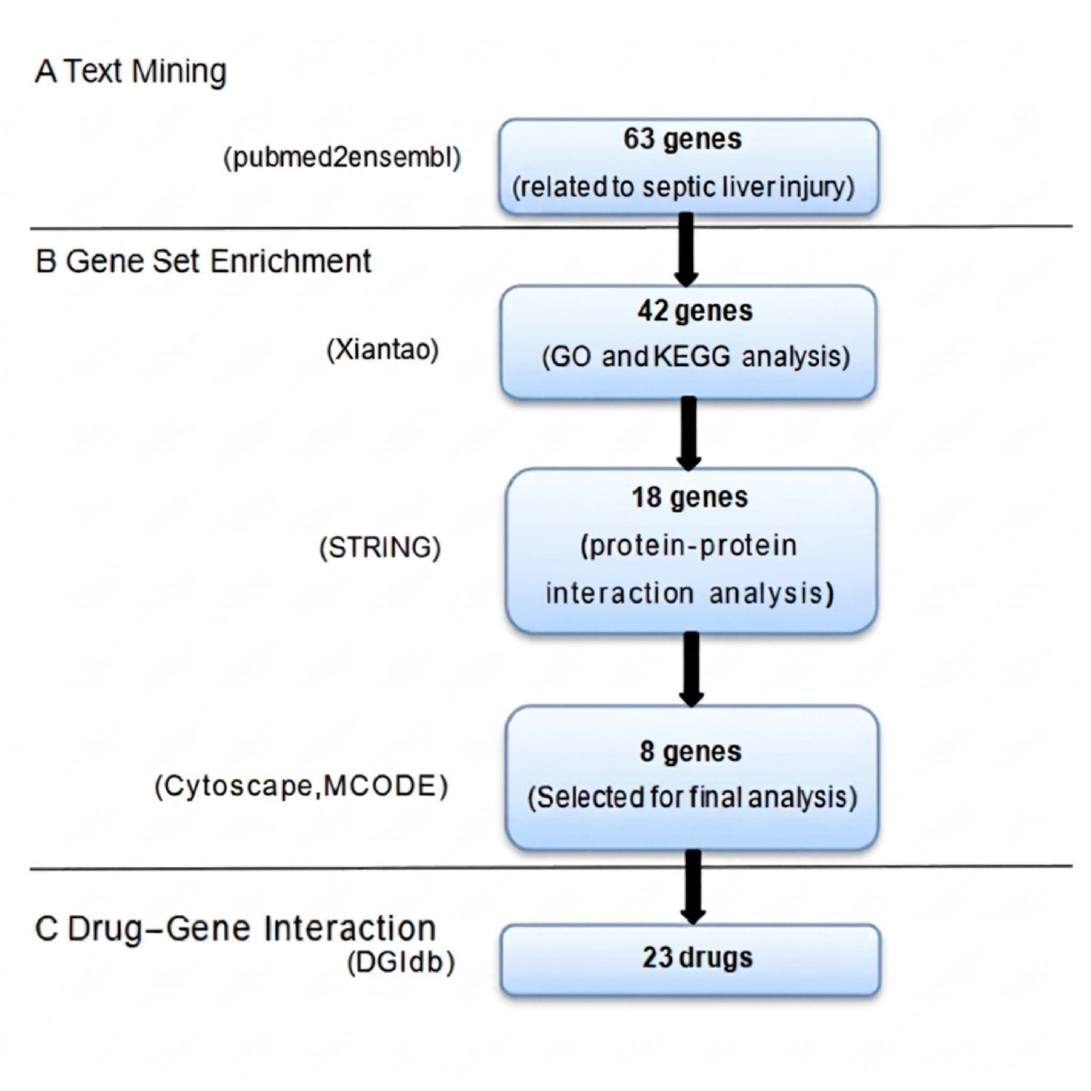
Summary of data mining results. (A) Text mining. Using the search term “septic liver injury” text mining was performed by pubmed2ensembl, and 63 genes were found. (B) Gene set enrichment. GO and KEGG pathway analyses were performed using Xiantao, and 42 genes were obtained. Next, using STRING and Cytoscape, 18 important genes were identified as hub genes, and 8 genes were selected for final analysis. (C) Drug-gene interactions: the final 8 genes were analyzed using the DGIDB, and 23 drugs were selected with therapeutic potential for septic liver injury. GO = gene ontology, KEGG = Kyoto encyclopedia of genes and genomes.

A schematic flowchart illustrating the overall bioinformatic analysis strategy is presented in Figure 1.

## 3. Results

### 3.1. Identification of differentially expressed genes

**Figure 2.**
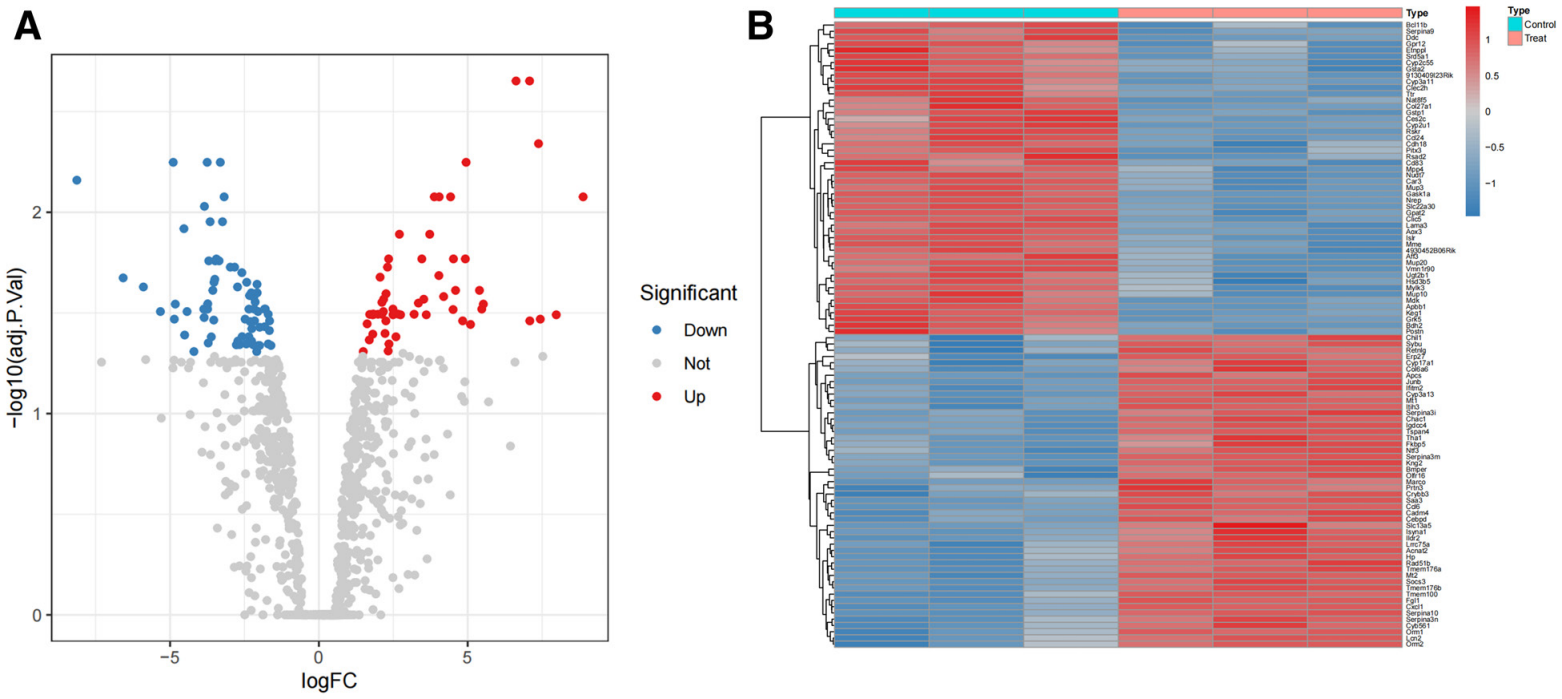
Analysis of DEGs. (A) Volcano plot of DEGs based on GSE226278. The red dots represent significantly up-regulated genes (log_2_FC >1, *P*-adj <.05), the blue dots represent significantly down-regulated genes (log_2_FC <−1, *P*-adj <.05), and the gray dots represent nonsignificant genes. (B) Heatmap of DEGs based on GSE226278 (|logFC| >1, *P*-adj <.05). DEGs = differential genes.

In this study, a sepsis liver injury gene expression profile GSE226278 was screened from the GEO database. This dataset comprises 3 samples from mice with septic liver injury and 3 control samples from healthy mice. DEGs were identified using a stringent cutoff of *P* <.05 and |logFC| >1. Our analysis revealed a total of 126 DEGs within the dataset, with 56 being upregulated and 70 downregulated (Fig. 2).

### 3.2. Results of text mining

We identified 63 genes associated with septic liver injury, as detailed in Table 1.

**Table 1:** Results of text mining.

SOAT1, SELP, CD160, PHGDH, SST, KNG1, GCG, IL1B, SLC25A10, IL-18, CASP1, LEP, CNR1, ABO, DYNLL1, SLC17A5, NOS1, LPO, GSTA1, CA5A, GAL, IGF1, GSN, TLR4, AGRP, IFNG, TAPBP, GPT, TNFRSF1B, CSF3, THBD, CDKN2A, HMOX1, MB, IL-6, CYCS, GAS6, NFKB1, PPARG, NOS2, EGF, ANXA5, KRT18, LBP, CD14, CYP1A2, TNF, EDA, TF, MMP9, CXCL8, RETN, ALB, GC, ITGB2, TP53, SHBG, SLC2A4, A2M, C1R, HMGB1, APP, TTR

A2M = alpha-2-macroglobulin, ABO = alpha 1-3-N-acetylgalactosaminyltransferase and alpha 1-3-galactosyltransferase, AGRP = agouti related neuropeptide, ALB = albumin, ANXA5 = annexin A5, APP = amyloid beta precursor protein, C1R = complement C1r subcomponent, CA5A = carbonic anhydrase 5A, CASP1 = caspase 1, CD14 = CD14 molecule, CD160 = CD160 molecule, CDKN2A = cyclin dependent kinase inhibitor 2A, CNR1 = cannabinoid receptor 1, CSF3 = colony-stimulating factor 3, CXCL8 = C-X-C motif chemokine ligand 8, CYCS = cytochrome c, CYP1A2 = cytochrome P450 family 1 subfamily A member 2, DYNLL1 = dynein light chain LC8-type 1, EDA = ectodysplasin A, EGF = epidermal growth factor, GAL = galanin and GMAP prepropeptide, GAS6 = growth arrest specific 6, GCG = glucagon, GCGC vitamin D binding protein, GPT = glutamic-pyruvic transaminase, GSN = gelsolin, GSTA1 = glutathione S-transferase alpha 1, HMGB1 = high mobility group box 1, HMOX1 = heme oxygenase 1, IFNG = interferon gamma, IGF1 = insulin like growth factor 1, IL-18 = interleukin-18, IL1B = interleukin 1 beta, IL-6 = interleukin-6, ITGB2 = integrin subunit beta 2, KNG1 = kininogen 1, KRT18 = keratin 18, LBP = lipopolysaccharide binding protein, LEP = leptin, LPO = lactoperoxidase, MB = myoglobin, MMP9 = matrix metallopeptidase 9, NFKB1 = nuclear factor kappa B subunit 1, NOS1 = nitric oxide synthase 1, NOS2 = nitric oxide synthase 2, PHGDH = phosphoglycerate dehydrogenase, PPARG = peroxisome proliferator activated receptor gamma, RETN = resistin, SELP = selectin P, SHBG = sex hormone binding globulin, SLC17A5 = solute carrier family 17 member 5, SLC25A10 = solute carrier family 25 member 10, SLC2A4 = solute carrier family 2 member 4, SOAT1 = sterol O-acyltransferase 1, SST = somatostatin, TAPBP = TAP binding protein, TF = transferrin, THBD = thrombomodulin, TLR4 = toll like receptor 4, TNF = tumor necrosis factor, TNFRSF1B = TNF receptor superfamily member 1B, TP53 = tumor protein p53, TTR = transthyretin.

### 3.3. Results of GO biological process and KEGG pathway analyses

**Figure 3.**
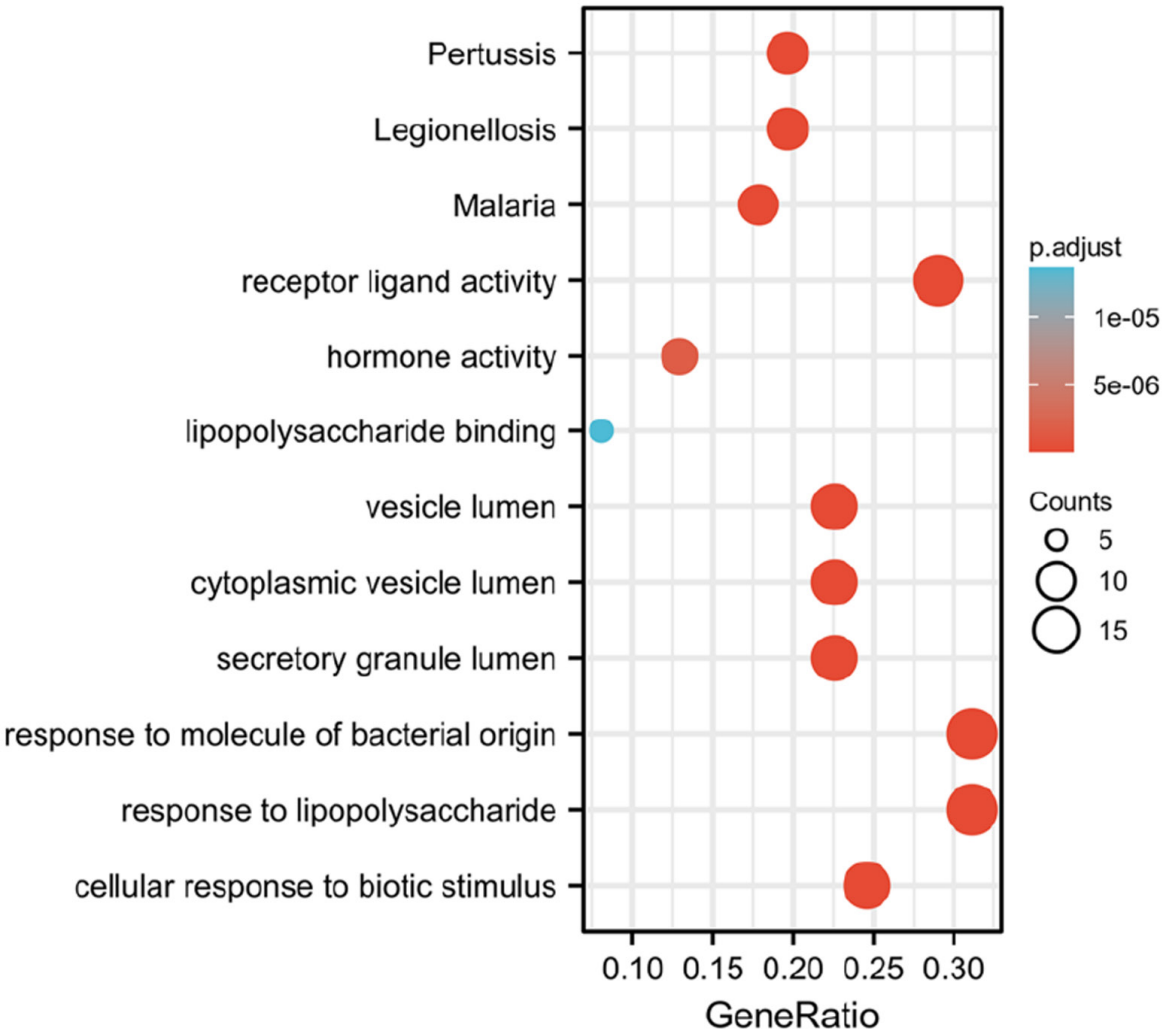
Enrichment analysis of genes in septic liver injury. The plot shows the top significantly enriched terms from both KEGG pathway analysis (top 3 terms) and GO analysis (subsequent terms). The GeneRatio indicates the proportion of input genes associated with each term, the dot size represents the number of genes, and the color intensity corresponds to the statistical significance (adjusted *P*-value). GO = gene ontology, KEGG = Kyoto encyclopedia of genes and genomes.

Utilizing Xiantao, we conducted a GO biological process analysis of the 63 genes, identifying terms significantly associated with septic liver injury pathology. To prioritize the most relevant annotations, a stringent *P*-value cutoff of .01 was applied. The top 5 biological processes with the highest enrichment scores include lipopolysaccharide (adjusted *P*-value = 1.33 × 10^−15^), response to bacterial molecules (1.38 × 10^−15^), cellular response to biotic stimuli (8.88 × 10^−13^), cellular response to lipopolysaccharide (2.49 × 10^−12^), and cellular response to molecules of bacterial origin (2.76 × 10^−12^). Additional significant biological processes encompassed regulation of interleukin-8 production, insulin secretion, lipid storage, smooth muscle cell proliferation, neuroinflammatory responses, hypoxia response, leukocyte migration, and STAT protein tyrosine phosphorylation regulation (Fig. 3, Table 2).

**Table 2:** Significantly enriched GO terms and KEGG pathways of genes.

Ontology	ID	Description	GeneRatio	BgRatio	*P*-value	*P*. adjusted	qvalue
BP	GO:0032496	Response to lipopolysaccharide	19/61	330/18,670	4.53 × 10^−19^	1.33 × 10^−15^	6.46 × 10^−16^
BP	GO:0002237	Response to molecule of bacterial origin	19/61	343/18,670	9.36 × 10^−19^	1.38 × 10^−15^	6.68 × 10^−16^
BP	GO:0071216	Cellular response to biotic stimulus	15/61	236/18,670	9.06 × 10^−16^	8.88 × 10^−13^	4.31 × 10^−13^
BP	GO:0071222	Cellular response to lipopolysaccharide	14/61	205/18,670	3.39 × 10^−15^	2.49 × 10^−12^	1.21 × 10^−12^
BP	GO:0071219	Cellular response to molecule of bacterial origin	14/61	212/18,670	5.41 × 10^−15^	2.76 × 10^−12^	1.34 × 10^−12^
CC	GO:0034774	Secretory granule lumen	14/62	321/19,717	9.97 × 10^−13^	1.54 × 10^−10^	1.29 × 10^−10^
CC	GO:0060205	Cytoplasmic vesicle lumen	14/62	338/19,717	2.00 × 10^−12^	1.54 × 10^−10^	1.29 × 10^−10^
CC	GO:0031983	Vesicle lumen	14/62	339/19,717	2.09 × 10^−12^	1.54 × 10^−10^	1.29 × 10^−10^
CC	GO:0031091	Platelet alpha granule	8/62	91/19,717	4.15 × 10^−10^	2.29 × 10^−08^	1.92 × 10^−08^
CC	GO:0031093	Platelet alpha granule lumen	7/62	67/19,717	1.61 × 10^−09^	7.11 × 10^−08^	5.96 × 10^−08^
MF	GO:0048018	Receptor ligand activity	18/62	482/17,697	3.04 × 10^−14^	9.33 × 10^−12^	6.59 × 10^−12^
MF	GO:0005179	Hormone activity	8/62	122/17,697	1.00 × 10^−08^	1.54 × 10^−06^	1.09 × 10^−06^
MF	GO:0001530	Lipopolysaccharide binding	5/62	35/17,697	1.34 × 10^−07^	1.37 × 10^−05^	9.69 × 10^−06^
MF	GO:0020037	Heme binding	7/62	135/17,697	4.46 × 10^−07^	3.42 × 10^−05^	2.42 × 10^−05^
MF	GO:0046906	Tetrapyrrole binding	7/62	145/17,697	7.23 × 10^−07^	4.44 × 10^−05^	3.14 × 10^−05^
KEGG	hsa05134	Legionellosis	11/56	57/8076	9.15 × 10^−14^	1.73 × 10^−11^	9.34 × 10^−12^
KEGG	hsa05144	Malaria	10/56	50/8076	9.18 × 10^−13^	8.68 × 10^−11^	4.69 × 10^−11^
KEGG	hsa05133	Pertussis	11/56	76/8076	2.57 × 10^−12^	1.62 × 10^−10^	8.76 × 10^−11^
KEGG	hsa05146	Amoebiasis	10/56	102/8076	1.45 × 10^−09^	6.86 × 10^−08^	3.71 × 10^−08^
KEGG	hsa05152	Tuberculosis	12/56	180/8076	2.48 × 10^−09^	9.36 × 10^−08^	5.06 × 10^−08^

GO = gene ontology, KEGG = Kyoto encyclopedia of genes and genomes.

In the process of KEGG pathway enrichment analysis, the adjusted *P*-value cutoff was set at .05. The 5 most significantly enriched pathways are the AGE-RAGE signaling pathway in Legionellosis (adjusted *P*-value = 1.73 × 10^−11^), Malaria (8.68 × 10^−11^), Pertussis (1.62 × 10^−10^), Amoebiasis (6.86 × 10^−08^), and Tuberculosis (9.36 × 10^−08^) (Fig. 3, Table 2).

### 3.4. Results of protein–protein interactions

**Figure 4.**
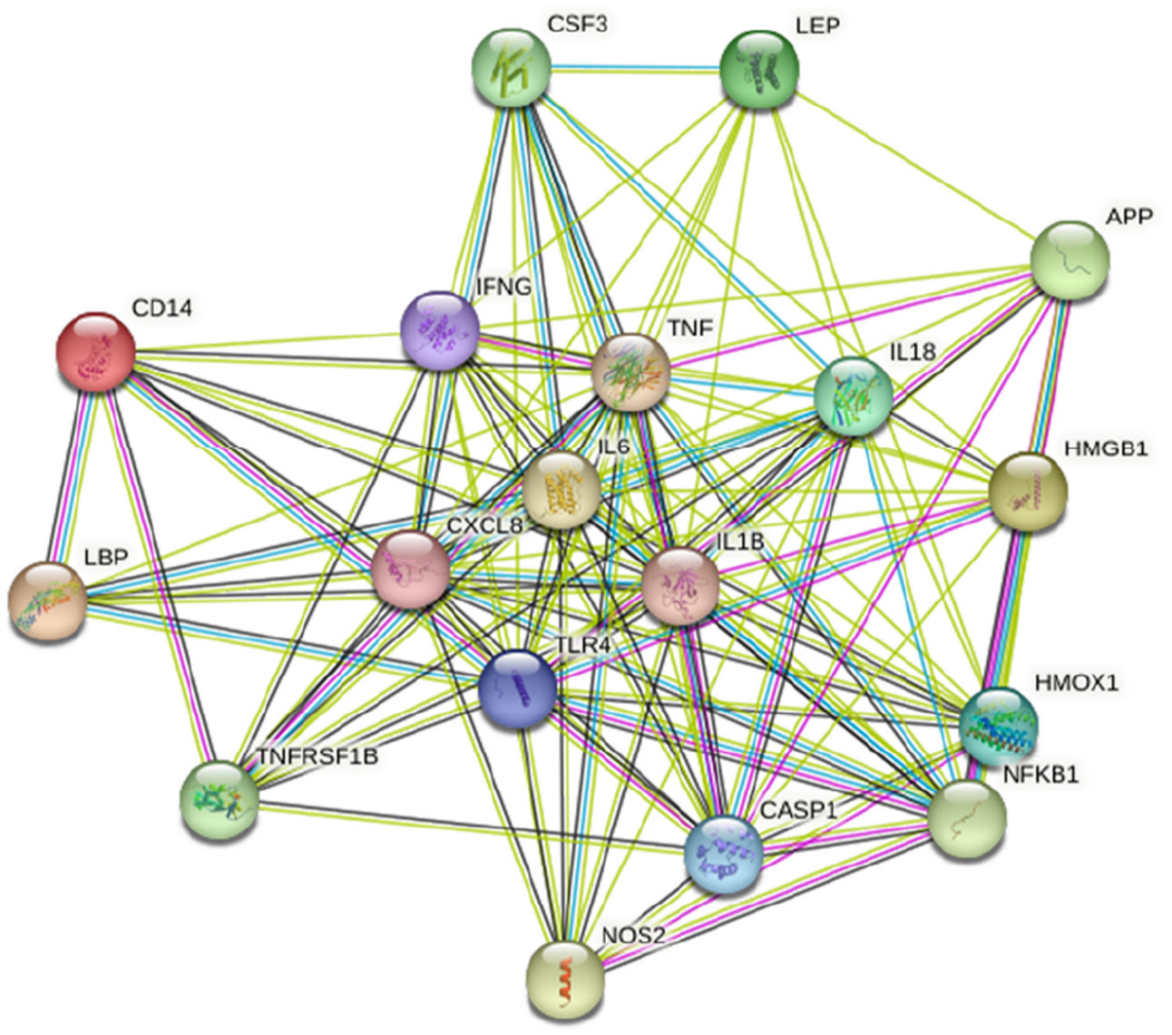
PPI network. The protein-protein medium (confidence score 0.400) interaction network of the targeted genes, was produced using STRING. The connecting line color indicates the types of interaction evidence with the confidence score set at 90%.

**Figure 5.**
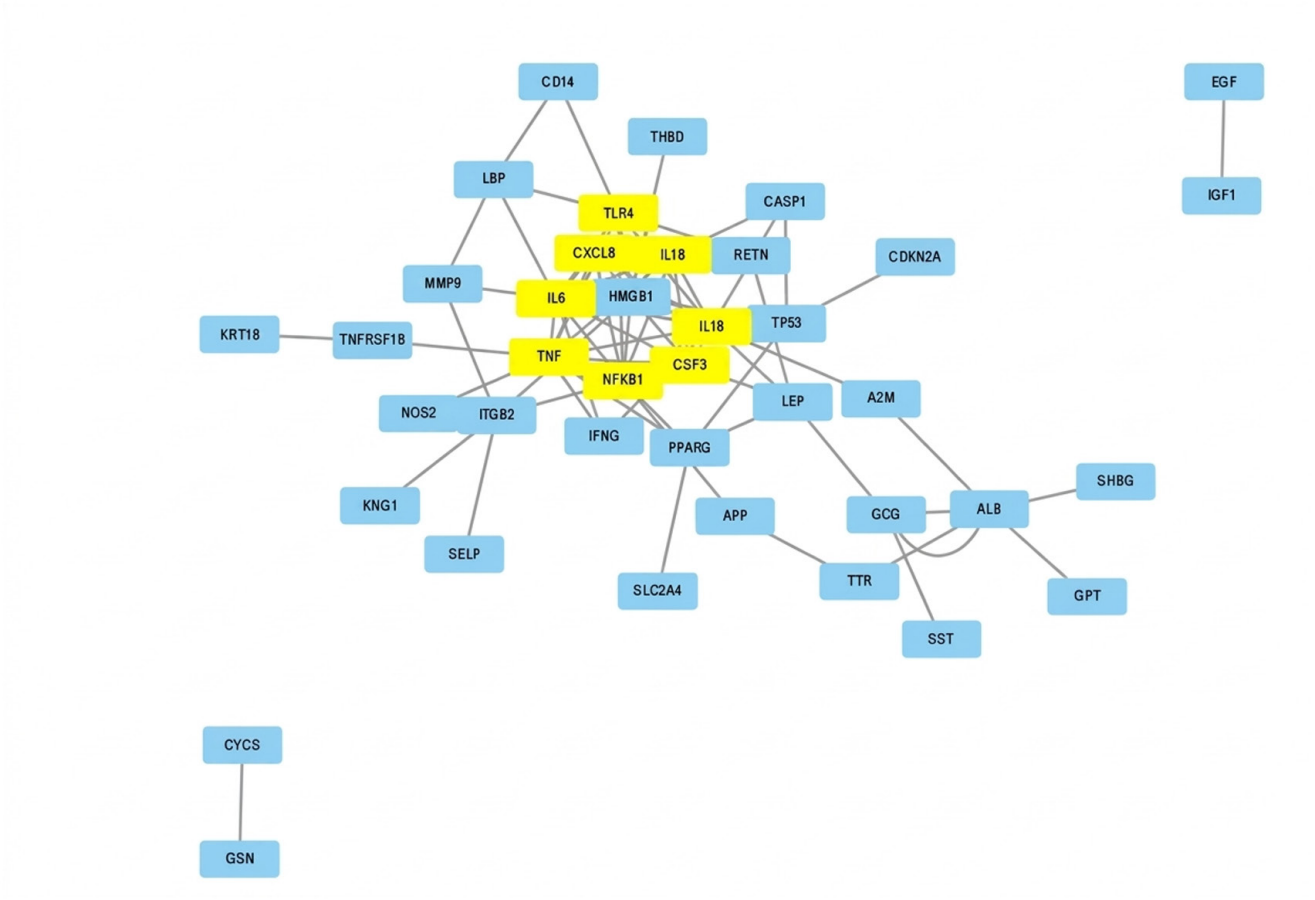
Gene module. Significant gene module (K-core 4). The yellow ones are the hub genes.

STRING was used to explain the protein–protein interaction network of target genes (Fig. 4). Data were subsequently imported into Cytoscape from the STRING EXPORT interface in “.tsv” format. Utilizing the MCODE application, we established a K-Core value of 4, while retaining other parameters at their default values. Consequently, MCODE identified a significant module comprising 8 pivotal genes: TLR4, CXCL8, IL-18, IL-6, IL1B, Tumor necrosis factor (TNF), NFKB1, and colony-stimulating factor 3 (CSF3), as illustrated in Figure 5. Hence, these genes were selected for further investigation.

### 3.5. Functional analysis of hub genes

**Figure 6.**
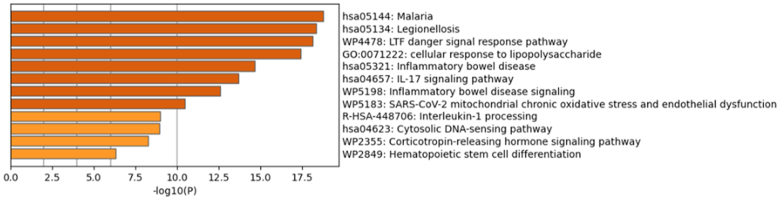
Bar graph of enriched terms across input gene lists, colored by *P*-values.

Consistent with our hypothesis, Metascape-facilitated functional annotation revealed that the pivotal genes predominantly regulate pathways associated with malaria, legionellosis, and the cellular response to lipopolysaccharide (Fig. 6)

### 3.6. Results of drug-gene interactions

We performed an in-depth analysis of drug-gene interactions for TLR4, CXCL8, IL-18, IL-6, IL1B, TNF, NFKB1, and CSF3, utilizing the DGIdb. Following a comprehensive review of the scientific literature, we identified 23 drugs with potential therapeutic applications for septic liver injury, as detailed in Table 3.

**Table 3:** Candidate drugs targeting genes for septic liver injury.

Number	Drug	Gene	Query score	Interaction score
1	ERITORAN	TLR4	17.22	22.48
2	RESATORVID	TLR4	8.61	11.24
3	ABX-IL-8	CXCL8	8.61	2.21
4	FOSCARNET	CXCL8	8.61	2.21
5	TALC	CXCL8	8.61	2.21
6	MEDI-2338	IL-18	8.61	17.67
7	GSK-1070806	IL-18	8.61	17.67
8	IBOCTADEKIN	IL-18	4.3	8.83
9	SILTUXIMAB	IL-6	17.22	9.89
10	OLOKIZUMAB	IL-6	17.22	9.89
11	CLAZAKIZUMAB	IL-6	12.91	7.42
12	PF-04236921	IL-6	8.61	2.47
13	SIRUKUMAB	IL-6	4.3	2.47
14	ELSILIMOMAB	IL-6	4.3	2.47
15	COR-001	IL-6	4.3	2.47
16	CANAKINUMAB	IL-1β	25.82	10.03
17	RILONACEPT	IL-1β	8.61	3.34
18	GEVOKIZUMAB	IL-1β	8.61	3.34
19	TT-301	IL-1β	8.61	3.34
20	GOLIMUMAB	TNF	21.52	4.55
21	PLACULUMAB	TNF	12.91	2.73
22	EFLAPEGRASTIM	CSF3	4.3	15.46
23	BENEGRASTIM	CSF3	4.3	15.46

CSF3 = colony-stimulating factor 3, CXCL8 = C-X-C motif chemokine ligand 8, IL-18 = interleukin 18, IL-1β = interleukin 1 beta, IL-6 = interleukin-6, NFKB1 = nuclear factor kappa B subunit 1, TLR4 = toll like receptor 4, TNF = tumor necrosis factor.

## 4. Discussion

The liver plays a pivotal role in maintaining the immune and metabolic equilibrium during sepsis,^[9]^ capable of engulfing invasive pathogens, producing acute-phase proteins, and regulating cytokine release, thereby orchestrating an adaptive inflammatory response and immune defense.^[10]^ However, in the context of systemic infection, the sepsis-induced cytokine storm and endotoxin production can inflict hepatocellular damage, precipitating organ dysfunction. This hepatocellular damage triggers the release of injury-associated molecular patterns, exacerbating the systemic inflammatory response and potentially leading to fatal outcomes in severe cases.^[11]^ Therefore, the identification of effective prognostic biomarkers is imperative for clinicians to detect septic liver injury and facilitate timely, informed medical interventions.^[12]^ In this study, we have delineated 8 target genes and their corresponding 23 therapeutic agents, all of which are implicated in 3 principal signaling pathways associated with septic liver injury, offering a comprehensive view of potential therapeutic targets.

In our study, we identified 8 key genes associated with septic liver injury: TLR4, CXCL8, IL-18, IL-6, IL1B, TNF, NFKB1, and CSF3, which play integral roles in the signaling pathways of malaria, legionellosis, and the cellular response to lipopolysaccharide. These genes are implicated in the regulation of these pathways and are targeted by a range of potential therapeutic agents. Our analysis has highlighted a series of drugs, such as ERITORAN, RESATORVID, ABX-IL-8, FOSCARNET, TALC, MEDI-2338, GSK-1070806, IBOCTADEKIN, and a suite of biologics like SILTUXIMAB, OLOKIZUMAB, CLAZAKIZUMAB, and others, which may modulate these pathways and offer novel treatment strategies for septic liver injury.

Toll like receptors are an important class of protein molecules involved in nonspecific immunity (innate immunity) and serve as a bridge between nonspecific and specific immunity. TLR4 can recognize Gram-negative bacterial lipopolysaccharides (LPS) and also recognize heat shock proteins released by host necrotic cells. Chen et al found that the silencing of TLR4 reduces LPS-induced liver injury by inhibiting inflammation and apoptosis through the TLR4/MyD88/NF-κB signaling pathway. TLR4 deficiency may protect the liver from LPS-induced ALI by inhibiting macrophage inflammation and apoptosis.^[13]^ From this perspective, TLR4 may be a potential therapeutic target for sepsis-induced liver injury.

The chemokine receptor CXCR1/2 and its ligand CXCL8 are crucial for the activation and transport of inflammatory mediators, as well as the progression and metastasis of tumors.^[14]^ In the past 2 decades, several small molecule CXCR1/2 inhibitors, CXCL8 release inhibitors, and neutralizing antibodies targeting CXCL8 and CXCR1/2 have been reported. As a single drug, such inhibitors are expected to be effective against various inflammatory diseases. However, currently, there is no research on the relationship between CXCL8 and sepsis-induced liver injury. Perhaps, our article has the potential to open a new chapter in CXCL8 research.

Interleukin-18 (IL-18, also known as interferon gamma inducible factor) is a protein encoded by the IL-18 gene in humans. The protein encoded by this gene is a pro-inflammatory cytokine. Many cell types, including hematopoietic and non-hematopoietic cells, have the potential to produce IL-18. Ono S, et al found that interleukin-12 and -18 induce severe liver damage in mice with sepsis.^[15]^ Sakao Y et al found that IL-18-deficient mice exhibit resistance to endotoxin-induced liver injury but are highly sensitive to endotoxin shock.^[16]^ It can be seen that IL-18 may play a key role in sepsis-induced liver injury.

Interleukin-6 (IL-6) is a multifunctional cytokine that plays an important role in host defense by regulating immune and inflammatory responses. IL-6 can diagnose early inflammation and warn of sepsis more quickly. In addition, the half-life of IL-6 is shorter than that of CRP and PCT, which can reflect the effect of antibiotic treatment faster and better reflect the prognosis of patients. Meanwhile, IL-6 can serve as a biomarker for disease severity and prognosis in cytokine storms, with its expression superior to TNF-α and IL-1.^[17]^ Numerous research findings indicate that IL-6 is a suitable target molecule for septic liver injury.

IL-1β works in an inflammatory environment by activating the plasma membrane receptor IL-1R1 expressed in various cell types, including endothelial cells, in a paracrine and autocrine manner. Research has shown that IL-1β disrupts vascular integrity, indicating its involvement in vascular injury.^[18]^ We showed that the IL-1β signaling pathway in endothelial cells inhibits VE-cadherin transcription by inactivating the transcription factor cAMP response element binding. In addition, gene repair of endothelial cAMP response element binding expression and transcriptional activity in mouse lung endothelial cells is sufficient to reverse sepsis-induced inflammatory lung injury, indicating a potential therapeutic target.^[19]^ However, the relationship between IL1B and sepsis-induced liver injury is currently unclear, and it may be a potential therapeutic target similar to sepsis-induced lung injury.

TNF is a small molecule protein secreted by macrophages. TNF is a multifunctional cytokine that plays an important role in the balance of the body and the pathogenesis of diseases. The TNF receptor signaling model has been extended to include linear ubiquitination and the formation of different signaling complexes associated with different functional outcomes such as inflammation, apoptosis, and necroptosis. Our understanding of TNF-induced gene expression has been enriched by the discovery of epigenetic mechanisms and concepts related to cell initiation, tolerance, and induction of “short-term transcriptional memory.”^[20]^ At present, there are few articles that solely study the relationship between TNF and sepsis-induced liver injury. Most of them use TNF as an inflammatory indicator to explore the relationship between other factors or drugs and the disease.

The (NFKB1) (p105/p50) subunits are important regulatory factors of NF-κ B in vivo activity. These effects are not limited to serving as dimeric partners for other NF - κ B subunits. On the contrary, the p50 homodimer plays a crucial role as an inhibitor of NF - κ B response, while the p105 precursor has multiple NF - κ B-independent functions.^[21]^ The study on cultured Nfkb1-/- microglia has demonstrated this, with reduced levels of pro-inflammatory genes and increased levels of anti-inflammatory genes expressed in these microglia.^[22]^ However, in vivo brain injury studies conducted in the same laboratory have shown that after injecting LPS into the hippocampus of Nfkb1-/- mice, there is an increase in leukocyte infiltration and expression of pro-inflammatory cytokines.^[22]^ There are currently few studies on the relationship between NFKB1 and sepsis-induced liver injury. Perhaps NFKB1 can become a new target for the treatment of sepsis-induced liver injury.

CSF3 is gradually recognized as an early diagnostic indicator of bacterial infectious diseases. ELISA is used to detect various bacterial infectious diseases, most of which show an increase in the level of CSF3 or the positive rate of detection. In diseases such as pneumonia, endocarditis, acute urinary tract infections, surgical infections, and neonatal sepsis, serum CSF3 levels are significantly elevated, often showing 2–3 times the normal reference value. Currently, there are no articles studying the relationship between CSF3 and sepsis-induced liver injury. Our study may provide a new perspective.

The drug candidates identified through our drug-gene interaction analysis primarily consist of novel agents or inhibitors. Among these, ERITORAN and RESATORVID are recognized as TLR4-targeting drugs. In murine models of chronic liver injury, ERITORAN has demonstrated a reduction in hepatic inflammation and fibrosis through the inhibition of the TLR4 signaling pathway.^[23]^ ERITORAN significantly suppressed the production of IL-1β, IL-6, IL-8, and IL-10, as well as TNF-α in whole blood assays. In animal models of sepsis, ERITORAN has been shown to ameliorate mortality rates and modulate cytokine production.^[24]^ RESATORVID, a specific small molecule inhibitor of TLR4 signaling, impedes the generation of lipopolysaccharide-induced inflammatory mediators through its interaction with the intracellular domain of TLR4. Restorvid, a selective TLR4 binder, disrupts the interaction between TLR4 and its co-factors, thereby inhibiting TLR4-mediated signal transduction and downstream events.^[25]^ The therapeutic agents targeting CXCL8, a key chemokine in inflammation, include ABX-IL-8, FOSCARNET, and TALC. ABX-IL-8 is a fully human IgG2 monoclonal antibody, generated through transgenic mouse technology, exhibiting high affinity and specificity for human interleukin-8.^[26]^ Preclinical evaluations have demonstrated that ABX-IL-8 suppresses tumor growth, angiogenesis, and metastasis in human melanoma models.^[27]^ Foscarnet serves as a viable option for both prophylactic and therapeutic interventions in patients undergoing hematopoietic stem cell transplantation. Foscarnet has demonstrated efficacy in treating a select cohort of patients with CMV-related gastrointestinal immune dysfunction, with over 67% showing improvement, as well as other infections.^[28,29]^

Talc, historically utilized in various industrial and consumer applications, is modern, purified, and graded talc preparations that are deemed safe and efficient for talc pleural fixation.^[30]^ Talc powder remains an affordable and accessible option, and despite some controversy, it continues to be a viable choice for pleural fixation.^[31]^ Drugs directed against IL-18 include MEDI-2338, GSK-1070806, and IBOCTADEKIN. In a Phase I clinical trial (NCT01322594), MEDI2338, an IL-18 monoclonal antibody, was administered to subjects with chronic obstructive pulmonary disease, with no reported serious adverse reactions, although pharmacodynamic data were lacking. GSK-1070806 is currently under evaluation in a Phase I clinical trial, with potential applications in kidney transplantation rejection, inflammatory bowel disease, Crohn disease, atopic dermatitis, and diabetes. IBOCTADEKIN has yet to advance to clinical trials.

The therapeutic agents directed against IL-6 include SILTUXIMAB, OLOKIZUMAB, CLAZAKIZUMAB, PF-04236921, SIRUKUMAB, ELSILIMOMAB, and COR-001. SILTUXIMAB, a chimeric human-mouse monoclonal antibody, is indicated for the treatment of Castleman disease.^[32]^ In patients with rheumatoid arthritis, the combination of OLOKIZUMAB and methotrexate demonstrates superior tolerability and efficacy compared to methotrexate monotherapy with a placebo.^[33]^ In a small group of patients with chronic active antibody-mediated rejection (cAMR), treatment with CLAZAKIZUMAB was associated with stabilization of eGFR and a reduction in DSA levels and graft inflammation.^[34]^ PF-04236921 has shown no significant advantage over placebo in the primary efficacy endpoint among patients with systemic lupus erythematosus.^[35]^ SIRUKUMAB is posited to exert therapeutic effects in lupus nephritis through its acute and localized impact on renal injury and modulation of aberrant B and T cell subsets in systemic lupus erythematosus patients.^[36]^ ELSILIMOMAB is infrequently utilized in clinical practice. COR-001 has not yet been implemented in clinical settings. Drugs targeting IL-1B include CANAKINUMAB, RILONACEPT, GEVOKIZUMAB, and TT-301. Canakinumab, a human monoclonal antibody against IL-1B, functions by neutralizing the IL-1B signal, thereby inhibiting inflammation in individuals with autoimmune diseases. This medication has received approval from the US Food and Drug Administration for treating familial cold autoinflammatory syndrome and Muckle-Wells syndrome. Its therapeutic potential is currently under investigation for a range of conditions, including rheumatoid arthritis, systemic juvenile idiopathic arthritis, chronic obstructive pulmonary disease, diabetes mellitus types 1 and 2, and ocular diseases.^[37]^ Recurrent pericarditis, a debilitating condition, is characterized by autoimmune inflammation primarily mediated by interleukin-1. Rilonacept, which blocks IL-1α and IL-1β signal transduction, has emerged as a valuable therapeutic option for recurrent pericarditis.^[38]^ The novel IL-1β-neutralizing antibody, gevokizumab, may ameliorate glycemic control and inflammation in patients with type 2 diabetes by modulating insulin production and activity.^[39]^ In murine models of acute nerve injury, TT-301 has demonstrated the capacity to modulate neuroinflammatory responses, leading to improved histological and functional outcomes.^[40]^ Drugs targeting TNF include GOLIMUMAB and PLACULUMAB. Golimumab, when administered subcutaneously, has been shown to elicit clinical responses, induce remission, promote mucosal healing, and enhance the quality of life in patients with active ulcerative colitis, as compared to placebo.^[41]^ PLACULUMAB is currently limited to use in scientific research. Eflapegrastim and benegrastim are therapeutic agents targeting CSF3. Eflapegrastim is characterized as a long-acting granulocyte colony-stimulating factor (G-CSF), featuring an IgG4 Fc fragment and a short polyethylene glycol linker. Eflapegrastim exhibits a prolonged bone marrow residence time compared to pegfilgrastim, consequently reducing the duration of neutropenia.^[42]^ BENEGRASTIM has not yet been applied in clinical practice.

This study has several limitations that should be acknowledged. First, the bioinformatic analysis of differentially expressed genes relied on a single microarray dataset (GSE226278). Although this dataset was rigorously selected for its relevance to septic liver injury and was supplemented with text-mining evidence to improve robustness, the inclusion of additional independent genomic datasets would enhance the generalizability and credibility of our findings. Future studies using multiple cohorts or multi-omics data are warranted to further validate the roles of the identified hub genes and pathways in septic liver injury. Moreover, although the hub genes identified here are central to inflammatory processes, their specific causal roles in septic liver injury require further experimental validation. Due to constraints in current experimental conditions and resources, we were unable to conduct functional validation in cellular or animal models. Subsequent studies employing techniques such as gene knockdown/knockout, targeted antagonism, or transgenic models are essential to definitively establish the mechanistic contributions of these genes to the pathogenesis of septic liver injury and to thoroughly evaluate the therapeutic potential of the proposed drug candidates. Additionally, the potential hepatotoxicity or additional metabolic burden that the candidate drugs may impose on a compromised liver remains unclear. Future preclinical and clinical studies should therefore carefully assess the safety and pharmacokinetic profiles of these therapeutic agents in the context of sepsis-induced liver dysfunction.

## 5. Conclusion

In this study, we have delineated a novel approach for the identification of candidate genes and associated drug targets implicated in septic liver damage. Our findings have identified a repertoire of 23 potential drugs that target 8 key genes, the majority of which have not been previously explored within the context of septic liver injury research. These discoveries lay the groundwork for future clinical trials and the emergence of precision medicine strategies for the treatment of septic liver injury.

## Author contributions

**Data curation:** Yan Yang.

**Formal analysis:** Jing Lv.

**Investigation:** Guobin Song.

**Methodology:** Jianfeng Chu.

**Supervision:** Shujun Sun.

**Writing – original draft:** Rui Chen.
